# Using Natural Language Processing in the LACE Index Scoring Tool to Predict Unplanned Trauma and Surgical Readmissions in South Africa

**DOI:** 10.1002/wjs.12523

**Published:** 2025-03-09

**Authors:** Umit Tokac, Jennifer Chipps, Petra Brysiewicz, John Bruce, Damian Clarke

**Affiliations:** ^1^ College of Nursing University of Missouri—Saint Louis Saint Louis Missouri USA; ^2^ Chair, Digital Health, Faculty of Community Health Sciences University of the Western Cape Cape Town South Africa; ^3^ School of Nursing & Public Health University of KwaZulu‐Natal Durban South Africa; ^4^ Department of Surgery University of KwaZulu‐Natal Durban South Africa; ^5^ Department of Surgery University of the Witwatersrand Johannesburg South Africa

**Keywords:** global surgery, outcomes, trauma

## Abstract

**Background:**

Unplanned and potentially avoidable readmission within 30 days post discharge is a major financial burden.

**Aim:**

To use text‐based electronic patient records to calculate the Charlson Comorbidity Index (CCI) score using a natural language processing technique to establish the feasibility and usefulness of the text‐based electronic patient records in identifying patients at risk for unplanned readmission.

**Methods:**

A retrospective review of electronic patient records for general and trauma surgery in a hospital in South Africa (2012–2022) was conducted using the LACE score. Validated sentiment analysis analyzed free text components of electronic patient records to compute the CCI score and to establish the feasibility and usefulness of the LACE score in identifying patients at risk for unplanned readmission.

**Results:**

Trauma surgery patients had a mean LACE score of 5.91 (SD = 2.41), with 8.44% scoring 10 or higher and a specificity and sensitivity of 91.63% and 13.81%, respectively. The general surgery patients had a mean LACE score of 7.75 (SD = 3.04), with 10.63% scoring 10 or higher and a specificity of 71.47% and a sensitivity of 44.80%, respectively. Logistic regression analysis revealed that LACE scores significantly predicted unplanned readmissions in both trauma (*β* = 0.11, *p* < 0.001; OR = 1.112, 95% CI [1.082, 1.143]) and general surgery (*β* = 0.15, *p* < 0.001; OR = 1.162, 95% CI [1.130, 1.162]) patients.

**Conclusion:**

The LACE score demonstrated the predictive value for readmission in trauma and general surgery patients. The LACE score was relatively effective in identifying patients who were less likely to be readmitted but showed limitations in identifying patients at higher risk of readmission. However, the successful use of natural language processing for data extraction of comorbidities shows promise on addressing the challenges around text‐based medical records.

## Graphical abstract

Unplanned and potentially avoidable readmission within 30 days post discharge is a financial burden. Using the LACE score to predict unplanned readmissions, natural language processing (NLP) was used to calculate the Charlson Comorbidity Index score. Although the LACE had poor sensitivity, NLP shows promise in addressing challenges around text‐based medical records.

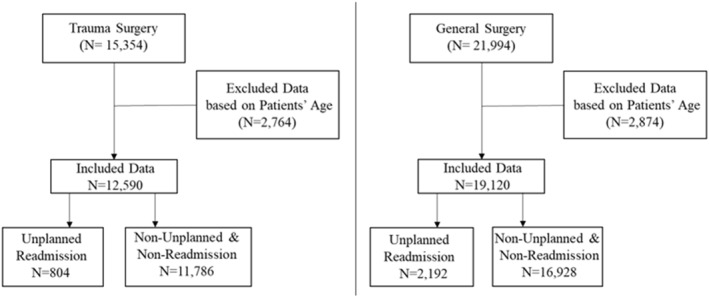

## 1 Introduction/Background

Unplanned and potentially avoidable readmissions within 30 days following discharge are a major financial burden on the health system [1, 2]. The global reported rates of unplanned readmission vary from 10% to 25%, with reported South African readmission rates of about 10% [2, 3]. Approximately 3% of the readmissions in general surgery are thought to be unplanned [4]. The major risk factors for unplanned readmission are premature discharge, number of comorbidities, nosocomial infections, adverse drug reactions, length of stay, and physician error [2, 5].

There is a need for a user‐friendly quantifiable risk assessment tool to assist clinicians to identify patients at risk for unplanned readmission [1, 4]. The LACE index scoring tool is one such tool based on acuity (elective vs. emergency admission), comorbidities, and emergency department consultations/visits in the last 6 months [1, 6]. The major limitation in using the LACE score is that the information required to calculate the score is often recorded in free text and needs to be manually extracted for data analysis.

The development of machine learning and the use of natural language processing (NLP) may provide a useful tool to analyze free text captured in electronic records. NLP is one of the main text‐mining machine‐learning techniques [7]. NLP is an artificial intelligence (AI) application in linguistics to make computers understand statements or words in human language [8]. NLP can extract deeper meaning structures from text through computational algorithms, which include features such as keyword counts and mapping analysis [7].

The aim of this study was to use NLP to analyze the free text components of a hybrid electronic medical registry (HEMR) for general surgery and trauma in South Africa, to proceed to calculate a LACE Index, and to identify patients at risk for unplanned readmission. In this study, patients who were readmitted 30 days were classified as unplanned readmissions.

## 2 Methods

### 2.1 Setting

The Department of Surgery at Grey's Hospital in Pietermaritzburg, KwaZulu‐Natal province, South Africa introduced the hybrid electronic medical registry (HEMR) in 2012 and integrated this system into the daily departmental workflow. It has been extensively reported in the literature and has provided data on morbidity and mortality since 2013. The HEMR captures demographic and physiological data and uses free text to document presenting symptoms, the clinical plan, operative notes, and discharge summaries [9].

## 3 Design

A retrospective review of unplanned readmissions for a general and trauma surgery population from 2012 to 2022 in HEMR was conducted. The LACE index score includes length of stay, number of emergency department visits, acuity of admission, and common comorbidities and was calculated for readmissions and nonreadmissions. NLP sentiment analysis was used to analyze the free text captured for the history of health problems and to calculate a Charlson Comorbidity Index (CCI) score.

### 3.1 Population and Sampling

**FIGURE 1:**
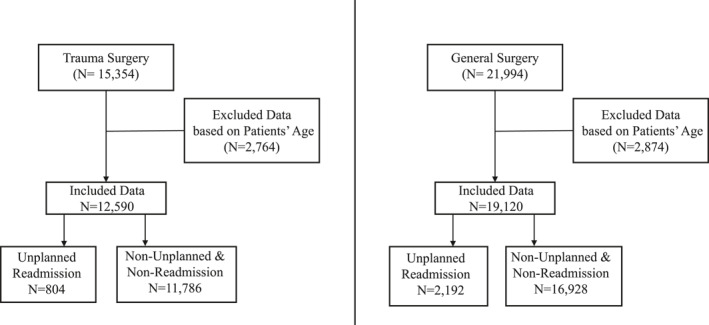
Flow chart for the sampling process of the dataset.

There were 15,354 trauma surgery patient records from 2012 to 2022 (804, 5.2% unplanned readmissions) and 21,994 general surgery patient records from 2012 to 2022 (2,192, 9.9% unplanned readmissions). Any patients aged younger than 18 or older than 100 years were excluded from the datasets. This sampling process is presented in Figure 1.

### 3.2 Calculating the LACE Index Score

The LACE index scoring tool has an overall total score ranging from 0 to 19 with a score of 10 and above indicating higher risk of readmission [1] Length of stay (score range 0–7), acuity of admission (score 0 or 3), and number of emergency department visits (score 0 or 4) were directly extracted from the database. The Charlson Comorbidity Index, which includes a consideration of age, (0–6) [10] was used to assign the severity of comorbidity (Appendix 1).

There was no variable for comorbidities. The free text, detailing the history of health complaints, was analyzed using NLP sentiment analysis for comorbidities scoring. Sentiment analysis identifies and extracts subjective information from text‐formed data in terms of keywords using a natural language processing (NLP) technique [11]. Sentiment analysis was conducted in R by creating a sentiment dictionary [12], which included weighted scores of health conditions (Table 1).

**TABLE 1:** The sentiment dictionary.

CCI health conditions	Weight
Myocardial infarction, CHF, PVD, CVA, dementia, COPD, PUD, mild liver disease, coronary artery disease, CAD, angina, coronary angioplasty, bypass surgery, CVD, CFF, congestive cardiac failure, stenting, gangrene, acute ischemia, aneurysm, stroke, TIA, transient ischemic attacks, OAD, obstructive airways disease, emphysema, cirrhosis, and hepatitis	+ 1
Moderate‐severe CKD, CA w/o mets DM with end‐organ damage, ESRD, end stage renal disease, renal disease, CLL, chronic lymphocytic leukemia, leukemia, MASS nonmalignant, carcinoma, CA, mass, carcinoma + malignant, diabetes with retinopathy, nephropathy, neuropathy, diabetic foot, DM, and diabetes + amputation	+ 2
Moderate liver disease, Severe liver disease, and Jaundice	+ 3
Metastatic solid CA, AIDS	+ 6
Each decade in age >40 years	+ 1

#### 3.2.1 Validation of Sentiment Analysis

The validity of AI sentiment scoring for patient comorbidity was evaluated by comparing AI‐generated sentiment scores with those produced by two clinical experts. Records from 52 randomly selected patients (26 trauma and 26 general surgery) were compared. Discrepancies in scoring were discussed and the coding for the NLP sentiment analysis was refined.

### 3.3 Data Analysis

Descriptive statistics, frequencies, and means were calculated for age, sex, length of stay (LOS), admission type, and the computed comorbidity score (Table 2). The LACE scoring tool was calculated for all unplanned readmission, nonunplanned readmission, and nonreadmitted patients, and data were tested for normality. Chi‐square and independent samples *t*‐test analyses were conducted to compare unplanned readmissions and nonunplanned and nonreadmission groups. Using logistic regression, unplanned readmitted (1) was used to identify the prediction of unplanned readmission by using the LACE score with odds ratio (OR 95% CI).

**TABLE 2:** Characteristics of unplanned readmission versus other patients for trauma surgery.

	Unplanned readmission (*N* = 804)	Other^a^ (*N* = 11,786)	Total (*n* = 12950)	Test	*p*‐value
Age	33.58 (± 11.78)	33.86 (± 12.26)	33.91 (± 12.26)	*T* = 0.654	*p* = 0.513
Sex	Female = 80 (10%), male = 724 (90%)	Female = 1814 (15%), Male = 9972 (85%)	Female = 1894 (15%) Male = 10696 (85%)	*X* ^2^ = 17.01	*p* < 0.001
LOS	*M* = 1.45 (± 2.08)	*M* = 1.85 (± 2.20)	*M* = 1.76 (±2.18)	*T* = 5.190	*p* < 0.001
Admission type	Elective = 50 (6%), Emergency = 747 (94%)	Elective = 619 (5%), Emergency = 10975 (95%)	Elective = 669 (5%), Emergency = 11722 (95%)	*X* ^2^ = 1.10	*p* = 0.295
Comorbidiy score	0.19, (± 0.6)	0.2 (± 0.65)	0.2 (± 0.65)	*T* = 0.67	*p* = 0.504
LACE score	6.59, (± 2.29)	5.93, (± 2.42)	5.91 (± 2.41)	*T* = 7.89	*p* < 0.001

^a^ Other = Planned readmissions & patients not readmitted.

## 4 Results

### 4.1 Demographics

The HEMR contains 15,354 records for trauma surgery (female = 2633, 17.15%; male = 12716, 82.82%; 5 patients with no reported sex), predominantly African (14,358, 93.51%), and ages ranging from 18 to 97 years (*M* = 33.91, SD = 12.29). A total of 804 unplanned readmissions (readmission rate 6.39%) were recorded. The HEMR also contains 21,994 general surgery records (female = 11495, 52.26%; male = 10450 47.51%; 49 patients with no reported sex), also predominantly African (16,990, 77.25%), with ages ranging from 18 to 98 (*M* = 49.33, SD = 17.44). A total of 2192 unplanned readmissions (readmission rate 6.39%) were recorded.

#### 4.1.1 Validation of Comorbidity Score

There was a concordance between AI and human assessments, with an 87% match rate. This high level of agreement indicates that the AI system's performance closely aligns with human expert judgment in identifying and evaluating patient comorbidities from the patient history of health complaints. The 13% discrepancy arose from differences in scoring approaches, with human experts including their interpretation in their scoring. These findings suggest that AI‐based sentiment analysis was sufficiently reliable for assessing patient comorbidities in this study.

#### 4.1.2 Characteristics of Unplanned Readmission vs Other Readmissions for Trauma Surgery

For trauma surgery, comparing patients' records of unplanned readmissions with other patients' records (planned readmissions and patients not readmitted), no significant differences in patients' age, admission type, and comorbidity score were found (Table 3). Patients with unplanned readmissions had significantly lower length of stay (LOS) (1.45, ± 2.08) compared to other patients (1.85, ± 2.20) (*T* = 5.19, *p* < 0.001), had a higher proportion of males (90% vs. 85%) (*X* ^2^ = 17.01, *p* < 0.001), and the LACE score was significantly higher (6.59, ± 2.29 vs. 5.93, ±2.42) (*T* = 7.89, *p* < 0.001).

**TABLE 3:** Characteristics of unplanned readmission versus nonunplanned and nonreadmitted patients for general surgery patients.

General surgery	Unplanned readmission (*N* = 2192)	Other* (*N* = 16,928)	Total (*n* = 19,120)	Test	*p*‐value
Age	*M* = 51.64 (± 16.15)	*M* = 49.49 (± 17.33)	*M* = 49.33 (± 17.44)	*t* = 5.81	*p* < 0.001
Sex	Female = 1144 (51%) Male = 1060 (49%)	Female = 9104 (53%), Male = 8033 (47%)	Female = 10248 (53%), Male = 9093 (47%)	*X* ^2^ = 1.12	*p* = 0.291
LOS	*M* = 3.32 (± 2.11)	*M* = 3.25 (± 1.97)	*M* = 3.04 (±2.08)	*t* = 1.52	*p* = 0.130
Admission type	Elective = 1360 (62%), Emergency = 831 (38%)	Elective = 8326 (49%), Emergency = 8602 (51%)	Elective = 9686 (51%), Emergency = 9563 (49%)	*X* ^2^ = 136.08	*p* < 0.001
Comorbidity score	*M* = 1.87 (± 1.84)	*M* = 1.53 (± 1.75)	*M* = 1.56 (± 1.76)	*t* = 8.22	*p* < 0.001
LACE score	*M* = 9.17, (± 3.09)	*M* = 7.82, (± 2.92)	*M* = 7.75 (± 3.04)	*t* = 19.38	*p* < 0.001

The average trauma surgery patients' LACE score for unplanned readmissions was 6.59 (± 2.29), and 1098 (8.44%) patients had scores of 10 and over, predicting being at risk of readmission. This indicated a specificity of 91.63% and a sensitivity of 13.81% of the LACE score. Similarly, the LACE score had a negative predictive value of 93.97% and positive predictive value of 10.11%. The predictive LACE score demonstrated an overall accuracy of 86.68% (95% CI: 86.07%, 87.27%). However, this accuracy should be interpreted with caution due to the imbalanced nature of the dataset, with the low prevalence of 8.7% of unplanned admissions.

#### 4.1.3 Characteristics of Unplanned Readmission vs Other Readmissions for General Surgery

For general surgery, there was no significant relationship between readmission to the hospital in 30 days and patient sex or patients' LOS (Table 3). The average age of patients with unplanned readmissions was significantly higher than the average age of patients with nonunplanned and nonreadmission (51.64, ± 16.15 vs. 49.49, ± 17.33) (*T* = 5.81, *p* < 0.001). Similarly, the comorbidity score for patients with unplanned readmissions (1.87, ± 1.84) was significantly higher than that of patients with other admissions (1.53, ± 1.75) (*T* = 8.22, *p* < 0.001), and the LACE score for patients with unplanned readmissions (9.17, ± 3.09) was significantly higher than that of patients with nonunplanned and nonreadmission (7.82, ± 2.92) (*T* = 19.38, *p* < 0.001).

The average general surgery patients' LACE score for unplanned readmissions was 9.17 (SD = 3.09), and 2228 (10.63%) patients had scores of 10 and over, predicting being at risk of readmission. This indicated a specificity of 71.47% and a sensitivity of 44.80% for the LACE score. Similarly, the LACE score had a negative predictive value of 90.91% and positive predictive value of 16.90%. The predictive LACE score demonstrated an overall accuracy of 68.4% (95% CI: 67.75%, 69.07%), which is slightly lower than the no‐information rate of 70.14%. However, this accuracy should be interpreted with caution due to the imbalanced nature of the dataset, with the low prevalence of 11.46% of unplanned admissions.

#### 4.1.4 Univariate Logistic Regression Trauma Surgery Unplanned Readmission

Logistic regression was conducted with readmission status as the dependent variable, LACE Score as the primary predictor, and sex as a covariate variable. The predictor variables, the LACE score (*β*_1_ = 0.11, SE = 0.01, *z* value = 7.59, *p* < 0.001), and sex (*β*_1_ = 0.52, SE = 0.12, *z* value = 4.31, *p* < 0.001) were found to contribute significantly to the trauma surgery model. Therefore, the LACE score predictor is associated with higher odds of an unplanned readmission (Exp (*β*_1_) = 1.114, 95% CI [1.084, 1.145]).

#### 4.1.5 Univariate Logistic Regression General Surgery Unplanned Readmission

Logistic regression was conducted with readmission status as the dependent variable, LACE Score as the primary predictor, and age as the covariate variable. The predictor variables, LACE Score (*β*_1_ = 0.16, SE = 0.008, *z* value = 19.05, and *p* < 0.001), and the predictor age (*β*_1_ = −0.004, SE = 0.001, *z* value = −2.58, and *p* < 0.01) were found to contribute significantly to the general surgery model. Therefore, the LACE score predictor is associated with higher odds of an unplanned readmission (Exp (β1) = 1.172, 95% CI [1.154, 1.191]).

## 5 Discussion

### 5.1 Use of NLP for Text Fields

Predicting the readmission risk is simpler for patients without complications; however, comorbidities like diabetes and cardiovascular diseases complicate accurate risk assessment [13]. In clinical practice, information on comorbidities is often captured as part of clinical notes on admission. This information is subsequently lost to the quantitative analysis of captured defined variables, which excludes clinical notes [14]. This can be addressed by using synoptic record reporting strategies to document specific data elements in tick box type entry formats. This should ensure data are captured directly into a usable and retrievable system. Despite this, there remains a tendency to not complete the requisite data capture sections and to rely on free text [15]. With the advent of natural language processing (NLP), there is now a potential to extract information on comorbidities from clinical notes, medical history, and discharge summaries [13]. A systematic review on machine learning applications in preventive health care has found that some machine learning‐driven applications can address inherent data deficiencies in health‐care datasets and provide models of interpretation that identifies significant risk factors associated with comorbidities [16]. Our study showed that AI‐based sentiment analysis was sufficiently reliable for assessing patient comorbidities in this setting. This confirms other findings in health care where the use of AI‐based sentiment analysis providing significant additional benefits over models without NLP and that NLP is effective in improving the accuracy of comorbidity calculations [13].

### 5.2 Accuracy of LACE Score as a Predictor

Our findings suggest that a considerable proportion of patients in both surgical groups had high LACE scores, indicating a higher likelihood of unplanned readmission. The comparison of patients' readmission records with LACE scores revealed interesting patterns. Among the other patients (nonunplanned and nonreadmission patients), high specificity was found which suggests that the LACE score was relatively effective in identifying patients who were less likely to be readmitted in both surgical groups. However, when examining the sensitivity of the LACE score to identify unplanned readmission patient records, sensitivity was low with only 13.7% of trauma surgery patients and 47.7% of general surgery patients having records that matched with the LACE score prediction. This finding confirms other studies of the low sensitivity of the LACE predicting readmissions of patients discharged from primary settings (sensitivity 42% (95% CI: 25%–59%) and specificity 79% (95% CI: 73%–85%) [17] and readmission of patients with medical conditions (LACE score ≥ 10 had sensitivity of 13%–26% and specificity of 89%–93% [18]. The limited predictive performance of the LACE score in this study suggests that its efficacy in identifying patients at higher risk of unplanned readmission may be suboptimal. However, this interpretation should be considered considering the study's limitations, particularly the imbalanced distribution of outcome events and the low prevalence of unplanned readmissions.

Logistic regression indicated that the LACE score significantly contributed to the prediction of patient readmission in both the trauma surgery model and the general surgery model. For trauma surgery, for every one‐unit increase in the LACE score, the odds of unplanned readmission increased by approximately 11.2%. Similarly, for general surgery, for every one‐unit increase in the LACE score, the odds of unplanned readmission increased by nearly 16.2%.

These findings indicate that the LACE score can be considered a significant predictor of unplanned readmission in both trauma and general surgery patients. However, it should be noted that the predictive value appears to be stronger for general surgery patients, as indicated by the higher odds ratio associated with the LACE score in the logistic regression analysis. The LACE score demonstrates some discriminative ability to identify patients not at risk of unplanned readmission; however, it faces significant challenges. This is due to the comparative low prevalence of unplanned readmissions, which can skew the model's performance in identifying patients at higher risk of unplanned readmission. This limitation is critical because accurately predicting positive cases (i.e., those who will have an unplanned readmission) is essential for effective intervention and resource allocation. To address these limitations, it is crucial to employ more robust evaluation methods. These could include testing different LACE thresholds taking into consideration the low incidence of unplanned readmissions, oversampling the unplanned readmissions or under sampling the other category, incorporating other clinically relevant variables, and leveraging machine learning and deep learning. This should enhance the predictive power of the model's ability to identify positive cases accurately.

## 6 Conclusion

The successful use of natural language processing and sentiment analysis for data extraction of comorbidities shows promise on addressing the challenges of text‐based medical records. However, the LACE score was relatively effective in identifying patients who were less likely to be readmitted in 30 days, while it showed limitations in identifying patients at higher risk of unplanned readmission. The discrepancy between the readmission records and LACE scores suggests the need for additional factors and assessments to improve the predictive accuracy of readmission risk in surgical patients. Future research should focus on developing more comprehensive risk assessment tools that integrate multiple factors and advanced AI technologies. These tools can provide a more nuanced understanding of unplanned readmission risk through the analysis of large datasets and identifying complex patterns that traditional statistical methods might miss. However, it is essential to recognize the limitations of AI, including the need for high‐quality data, the potential for algorithmic bias, and the importance of interpretability in clinical settings.

## Author Contributions

**Umit Tokac:** conceptualization, data curation, formal analysis, funding acquisition, investigation, methodology, project administration, software, validation, visualization, writing – original draft, writing – review and editing. **Jennifer Chipps:** conceptualization, data curation, funding acquisition, investigation, methodology, project administration, validation, visualization, writing – original draft, writing – review and editing. **Petra Brysiewicz:** conceptualization, investigation, methodology, validation, visualization, writing – original draft, writing – review and editing. **John Bruce:** conceptualization, data curation, investigation, methodology, validation, visualization, writing – original draft, writing – review and editing. **Damian Clarke:** conceptualization, data curation, investigation, methodology, resources, validation, visualization, writing – original draft, writing – review and editing.

## Ethics Statement

The study was conducted in accordance with the Declaration of Helsinki and approved by the Ethics Committee of the University of the Western Cape (HS22/4/6, 2022) and University of Missouri (IRB Review Number 351071).

## Conflicts of Interest

The authors declare no conflicts of interest.

## Appendix 1 LACE Index Scoring

**Table:**

			Score
L	Length of stay	1	1
		2	2
		3	3
		4–6	4
		7–13	5
		≥ 14	7
A	Acuity of admission	Elective	0
		Urgent/Emergent	3
C	Charlson Comorbidity Score	0	0
		1	1
		2	2
		3	3
		≥ 4	5
E	Emergency department visit last 6 months	0	0
		1	1
		2	2
		3	3
		≥ 4	4
LACE			Total
